# Beyond Foodborne Pathogens: An Integrated Assessment of the Microbiological Safety of Bulgarian White Brined Cheese

**DOI:** 10.3390/foods15162808

**Published:** 2026-08-11

**Authors:** Neli Ermenlieva, Sylvia Stamova, Sevginar Ibryamova, Nadezhda Ivanova, Petya Atanasova, Velichka Marinova, Gabriela Tsankova, Emilia Georgieva

**Affiliations:** 1 Department of Microbiology and Virusology, Faculty of Medicine, Medical University, 9000 Varna, Bulgaria; gabriela_sc@abv.bg; 2 Department of Pharmaceutical Chemistry, Faculty of Pharmacy, Medical University, 9000 Varna, Bulgaria; silvia.stamova@mu-varna.bg; 3 Faculty of Natural Sciences, Department of Biology, Shumen University, 9000 Shumen, Bulgaria; s.ibryamova@shu.bg; 4 Department of Pharmaceutical Technologies, Faculty of Pharmacy, Medical University, 9000 Varna, Bulgaria; nadejda.ivanova@mu-varna.bg; 5 Professor Dr. Asen Zlatarov Vocational High School of Tourism, 9000 Varna, Bulgaria; petya.atanasova@pgtvarna.com; 6 Department of Commodity Science, Faculty of Economics, University of Economics, 9000 Varna, Bulgaria; velichka.peewa@abv.bg; 7 Training Sector “Medical Laboratory Technician”, Medical College—Varna, Medical University, 9000 Varna, Bulgaria; emiliya.georgieva@mu-varna.bg

**Keywords:** white brined cheese, microbiological safety, artisanal cheese, foodborne pathogens, *Escherichia coli*, *Listeria monocytogenes*, *Salmonella* spp., yeast microbiota, MALDI–TOF MS, food hygiene

## Abstract

Background: White brined cheese represents one of the most important traditional fermented dairy products in Bulgaria. Its microbiological quality is commonly evaluated through the detection of foodborne pathogens; however, such an approach provides limited information regarding production hygiene and the natural microbial ecosystem of the product. Methods: The present study performed an integrated microbiological assessment of 100 Bulgarian white brined cow’s milk cheese samples (50 artisanal and 50 industrial) by simultaneously evaluating hygiene indicator microorganisms (*Escherichia coli* and coagulase-positive staphylococci), major foodborne pathogens (*Listeria monocytogenes* and *Salmonella* spp.), and the natural yeast microbiota using standardized ISO methods. Yeast species were identified by MALDI–TOF MS, and statistical analyses were performed using Fisher’s exact test and the Mann–Whitney U test. Results: *Listeria monocytogenes*, *Salmonella* spp., and coagulase-positive staphylococci were not detected in any cheese sample. *Escherichia coli* was detected in only two samples (2.0%), with no significant difference between artisanal and industrial cheeses (*p* > 0.05). In contrast, yeasts were detected in 99% of the samples, and nine yeast taxa were identified. Significant differences in yeast species distribution were observed between production systems, with *Torulaspora delbrueckii* predominating in artisanal cheeses and *Debaryomyces hansenii* in industrial products (*p* < 0.001). Conclusions: The results demonstrate that artisanal white brined cheese is not inherently associated with a higher microbiological risk when good manufacturing and hygienic practices are applied. More importantly, the study shows that combining foodborne pathogens, hygiene indicators, and indigenous yeast microbiota provides a substantially more comprehensive assessment of microbiological quality than pathogen detection alone, supporting an integrated concept for the evaluation of traditional fermented dairy products.

## 1. Introduction

White brined cheese is one of the most important traditional fermented dairy products in the Balkan Peninsula and the Eastern Mediterranean region [1]. In Bulgaria, it represents a product of considerable nutritional, economic, and cultural importance and occupies a central position in the national dairy sector [2]. The characteristic manufacturing technology, based on milk coagulation followed by ripening in brine, creates a unique environment that supports the development of a complex microbial community. Consequently, white brined cheese should be regarded not merely as a dairy product but as a dynamic microbial ecosystem in which technological microorganisms, spoilage microbiota, and potential foodborne pathogens coexist throughout production, ripening, storage, and distribution [3].

The microbial composition of white brined cheese is determined by numerous factors, including the microbiological quality of raw milk, hygienic conditions during manufacture, processing practices, storage conditions [4], and the effectiveness of food safety management systems. Beneficial microorganisms, particularly lactic acid bacteria, play a fundamental role in fermentation, product stabilization, and the inhibition of undesirable microorganisms. At the same time, other microbial groups may adversely affect product quality or represent potential hazards to consumer health [5]. Therefore, the microbiological safety of white brined cheese should be interpreted as the result of interactions among technological microbiota, hygiene indicator microorganisms, spoilage organisms, foodborne pathogens, and manufacturing practices rather than the isolated occurrence of individual microorganisms.

Comprehensive microbiological evaluation requires the simultaneous assessment of different microbial groups that provide complementary information regarding product safety and hygienic quality. Among them, *Escherichia coli* is widely recognized as an indicator of fecal contamination and inadequate hygienic practices during milk production and cheese manufacture [6,7]. Although most strains are harmless, their presence may indicate deficiencies in sanitation and an increased likelihood of contamination by enteric pathogens [8]. *Staphylococcus aureus* represents another microorganism of major concern because it may contaminate milk and dairy products through infected animals or human handling and, in the case of enterotoxigenic strains, produce heat-stable enterotoxins responsible for food poisoning [9,10,11]. Particular attention is also devoted to *Listeria monocytogenes* and *Salmonella* spp., two of the most significant foodborne bacterial pathogens worldwide. Their detection in ready-to-eat dairy products is of major public health concern and is subject to strict microbiological criteria established by food safety legislation [12,13,14].

In addition to bacterial hazards, yeasts constitute an important component of the microbiota of white brined cheeses. Depending on their species composition and abundance, they may contribute positively to ripening and sensory development or, conversely, promote spoilage through undesirable metabolic activities that shorten product shelf life and impair sensory quality. Consequently, the combined evaluation of bacterial pathogens, hygiene indicator microorganisms, and yeast populations provides a broader understanding of the microbiological status of white brined cheese than the investigation of individual microbial groups alone.

Although numerous studies have investigated individual microbiological hazards in dairy products, relatively few have simultaneously evaluated hygiene indicator microorganisms, major foodborne pathogens, and yeast populations within the same collection of artisanal and industrial white brined cheeses [15]. Furthermore, comprehensive microbiological data on Bulgarian white brined cow’s milk cheese remain limited despite the product’s considerable importance for the national dairy industry. Such integrated investigations are essential for evaluating microbiological safety, identifying potential sources of contamination, and supporting evidence-based quality assurance and food safety strategies.

The present study was intentionally restricted to cow’s milk cheese to minimize variability related to milk species and to allow a more direct comparison between artisanal and industrial production systems. Since the composition and indigenous microbiota of the raw material may influence the microbial ecology of the resulting cheese, extending this approach to white brined cheeses produced from sheep’s and goat’s milk represents a relevant direction for future research.

Therefore, the aim of the present study was to perform a comprehensive microbiological safety assessment of artisanal and industrial Bulgarian white brined cow’s milk cheese by evaluating hygiene indicator microorganisms, major bacterial foodborne pathogens, and yeast populations using standardized microbiological methods. The study provides an integrated overview of the microbiological status of this traditional dairy product and contributes new data supporting the improvement of its microbiological safety and quality.

## 2. Materials and Methods

### 2.1. Sample Collection

A total of 100 samples of Bulgarian white brined cow’s milk cheese were included in the present study. Of these, 50 samples originated from artisanal production and 50 samples from industrial production. The samples were collected over a one-year period to ensure representativeness with respect to different production batches.

Samples were classified according to their production and distribution context. Artisanal cheeses were obtained directly from small-scale dairy producers and farmers’ markets, with most producers operating in accordance with Bulgarian Ordinance No. 26 of 14 October 2010 [16]. Industrial cheeses were obtained from the retail market and represented products manufactured by established Bulgarian dairy companies.

Only samples labelled as white brined cow’s milk cheese were included in the study. Products were purchased either in their original commercial packaging or under documented artisanal production conditions. Samples with damaged packaging or visible signs of improper storage were excluded from the investigation.

Following purchase, all samples were transported to the laboratory under refrigerated conditions (4 ± 2 °C). Microbiological analyses were initiated within 24 h of sampling.

### 2.2. Sample Preparation

Sample preparation and all microbiological analyses were performed under aseptic laboratory conditions in accordance with the requirements of the relevant International Organization for Standardization (ISO) methods [17,18,19,20,21]. Maximum Recovery Diluent (MRD; Merck, Darmstadt, Germany) was used for the preparation of the initial sample suspension. Subsequent serial dilutions, cultivation conditions, and microbiological procedures were carried out according to the corresponding ISO standard for each target microorganism. All analyses were performed in triplicate.

### 2.3. Enumeration of β-Glucuronidase-Positive Escherichia coli

The enumeration of β-glucuronidase-positive *Escherichia coli* was performed in accordance with EN ISO 16649-2 [17]. Following sample preparation, appropriate decimal dilutions were surface-plated onto Tryptone Bile X-glucuronide (TBX) agar (HiMedia, Mumbai, India). After incubation under the conditions specified in the standard, characteristic blue-green colonies were enumerated, and the results were expressed as log_10_ CFU/g.

Where necessary, representative colonies were biochemically confirmed using Kligler Iron Agar (KIA) (BulBio, Sofia, Bulgaria) and the indole production test (BulBio, Sofia, Bulgaria).

### 2.4. Enumeration of Coagulase-Positive Staphylococci

The enumeration of coagulase-positive staphylococci was performed in accordance with EN ISO 6888-1 [18]. Appropriate decimal dilutions were surface-plated onto Baird–Parker agar (HiMedia, Mumbai, India) supplemented with egg yolk tellurite emulsion. Following incubation under the conditions specified in the standard, typical colonies were enumerated, and representative isolates were confirmed using the coagulase test (BulBio, Sofia, Bulgaria). Results were expressed as log_10_ CFU/g of cheese.

### 2.5. Detection of Salmonella spp. in the Food Chain

The detection of *Salmonella* spp. was performed in accordance with EN ISO 6579-1 [19]. Following non-selective pre-enrichment in Buffered Peptone Water (BPW) (HiMedia, Mumbai, India), the samples were subjected to selective enrichment in Rappaport–Vassiliadis Soya (RVS) broth (HiMedia, Mumbai, India) and Müller–Kauffmann Tetrathionate–Novobiocin (MKTTn) broth (HiMedia, Mumbai, India). After incubation, the enrichment cultures were streaked onto Xylose Lysine Deoxycholate (XLD) agar (HiMedia, Mumbai, India). Presumptive colonies were evaluated on the basis of their characteristic morphology on XLD agar and subsequently confirmed according to the identification scheme specified in EN ISO 6579-1, including serological confirmation using a specific *Salmonella* antiserum (BulBio, Sofia, Bulgaria). Results were expressed as the presence or absence of *Salmonella* spp. in 25 g of cheese.

### 2.6. Detection of Listeria monocytogenes in the Food Chain

The detection of *Listeria monocytogenes* was performed in accordance with EN ISO 11290-1 [20]. Following primary selective enrichment in Half Fraser broth (HiMedia, Mumbai, India), secondary enrichment was carried out in Fraser broth, followed by inoculation onto Agar Listeria according to Ottaviani and Agosti (ALOA) (HiMedia, Mumbai, India). Presumptive colonies were evaluated on the basis of their characteristic morphology and confirmed according to the procedures specified in the standard. Results were expressed as the presence or absence of *L. monocytogenes* in 25 g of cheese.

### 2.7. Enumeration and Species Identification of Yeasts

The enumeration of yeasts was performed in accordance with ISO 21527-1 [21]. Appropriate decimal dilutions were surface-plated onto Dichloran Rose Bengal Chloramphenicol (DRBC) agar (HiMedia, Mumbai, India) and incubated at 25 °C for 5 days. Following enumeration of the characteristic colonies, representative isolates were subcultured to obtain pure cultures.

Yeast species identification was performed using matrix-assisted laser desorption/ionization time-of-flight mass spectrometry (MALDI–TOF MS) with the Bruker Biotyper system, according to the methodology described in our previous study [22].

### 2.8. Criteria for the Evaluation of Microbiological Safety and Microbial Quality

The microbiological safety of the investigated cheese samples was evaluated by comparing the obtained results with the microbiological criteria established by Commission Regulation (EC) No. 2073/2005 on microbiological criteria for foodstuffs [23], including all subsequent amendments.

For microorganisms subject to regulatory microbiological criteria (*Listeria monocytogenes* and *Salmonella* spp.), compliance was assessed according to the requirements for ready-to-eat foods specified in the Regulation.

The results for coagulase-positive staphylococci were interpreted as indicators of production hygiene and the potential risk of staphylococcal enterotoxin production at elevated bacterial concentrations.

The counts of β-glucuronidase-positive *Escherichia coli* were used as indicators of production hygiene, raw material quality, and the effectiveness of good manufacturing and hygienic practices.

As no regulatory microbiological limits have been established for yeasts in white brined cheese, the obtained results were used to evaluate the microbial quality of the product, its technological characteristics, and its susceptibility to microbiological spoilage. Species identification of the isolated yeasts provided additional information on yeast diversity and the microorganisms involved in cheese ripening and storage.

### 2.9. Statistical Analysis

Statistical analyses were performed using IBM SPSS Statistics version 27.0 (IBM Corp., Armonk, NY, USA). Descriptive statistics were calculated for all quantitative variables and expressed as mean ± standard deviation (SD), median, interquartile range (IQR), minimum and maximum values, as appropriate.

The prevalence of positive samples and the distribution of yeast species between artisanal and industrial cheeses were compared using Fisher’s exact test, owing to the low expected frequencies in several contingency table cells.

Since the quantitative counts of presumptive *Staphylococcus* spp. did not follow a normal distribution and the sample size of positive samples was limited, differences in bacterial concentrations between artisanal and industrial cheeses were evaluated using the Mann–Whitney U test. All statistical tests were two-tailed, and differences were considered statistically significant at *p* < 0.05.

Microbiological counts were expressed as log_10_ colony-forming units per gram (log_10_ CFU/g) prior to statistical analysis.

## 3. Results

### 3.1. Occurrence of Escherichia coli as a Hygiene Indicator 

*Escherichia coli* was detected in two of the 100 analyzed white brined cow’s milk cheese samples (2.0%). One positive sample originated from artisanal production and one from industrial production, corresponding to a prevalence of 2.0% within each production group.

In the positive artisanal sample, nine characteristic turquoise colonies were observed on Tryptone Bile X-glucuronide (TBX) agar at the 10^−1^ dilution, whereas seven characteristic colonies were detected in the positive industrial sample at the same dilution. No typical *E. coli* colonies were observed in the remaining cheese samples.

Representative colonies were subjected to biochemical confirmation. The isolates showed positive indole production, negative oxidase reaction, and characteristic reactions on Kligler Iron Agar, demonstrating glucose and lactose fermentation with gas production and no hydrogen sulfide formation. These biochemical characteristics confirmed the presumptive isolates as *Escherichia coli*.

The distribution of positive samples according to the production system is presented in Table 1.

### 3.2. Detection and Quantification of Presumptive Staphylococcus spp.

Presumptive *Staphylococcus* spp. were detected in 49 of the 100 examined white brined cow’s milk cheese samples (49.0%). Positive samples were identified in 28 of the 50 artisanal cheeses (56.0%) and 21 of the 50 industrial cheeses (42.0%), whereas the remaining samples were negative (Table 2). Although presumptive staphylococci were isolated more frequently from artisanal cheeses, the difference in prevalence between artisanal and industrial products was not statistically significant (Fisher’s exact test, *p* = 0.230).

The quantitative characteristics of presumptive staphylococci isolated from positive samples are summarized in Table 3. Bacterial counts ranged from 2.00 to 3.74 log_10_ CFU/g in artisanal cheeses and from 2.00 to 3.91 log_10_ CFU/g in industrial cheeses. The mean bacterial concentration was 2.90 ± 0.55 log_10_ CFU/g for artisanal cheeses and 3.18 ± 0.42 log_10_ CFU/g for industrial cheeses. Median bacterial counts were 2.90 and 3.30 log_10_ CFU/g, respectively, with interquartile ranges (IQR) of 2.49–3.40 and 3.00–3.42 log_10_ CFU/g. Comparison of bacterial counts between the two production groups using the Mann–Whitney U test demonstrated no statistically significant difference (U = 214.0, *p* = 0.108).

To further characterize contamination levels, positive samples were classified according to bacterial concentration (Table 4). Among artisanal cheeses, bacterial counts were relatively evenly distributed across the lower and intermediate concentration ranges. In contrast, industrial cheeses were predominantly represented by samples containing 3.00–3.49 log_10_ CFU/g. Only a small proportion of positive samples in either production group exhibited bacterial counts ≥ 3.50 log_10_ CFU/g, indicating that high bacterial loads were uncommon regardless of the production type.

Confirmation of presumptive isolates is presented in Table 5. Representative colonies exhibiting typical morphology on Baird–Parker agar were selected from each positive sample and subjected to the plasma coagulase test according to BG ISO 6888-1. None of the tested isolates demonstrated plasma coagulation, indicating that no coagulase-positive staphylococci (*Staphylococcus aureus*) were confirmed among the investigated cheese samples. Consequently, all recovered isolates were classified as coagulase-negative staphylococci (CoNS).

Overall, the statistical analysis demonstrated that neither the prevalence nor the concentration of presumptive staphylococci differed significantly between artisanal and industrial cheeses. Although minor numerical differences were observed, these reflected the natural variability among individual cheese samples rather than systematic differences associated with the production type. The absence of plasma coagulase activity in all representative isolates further demonstrates that the detected staphylococci did not represent coagulase-positive foodborne pathogens, supporting the favourable microbiological safety status of both artisanal and industrial white brined cow’s milk cheeses with respect to this microbiological criterion.

### 3.3. Detection of Salmonella spp.

*Salmonella* spp. was not detected in any of the 100 white brined cow’s milk cheese samples analyzed. In seven of the 50 artisanal cheese samples, colonies with atypical morphology were observed on XLD agar. These colonies did not exhibit the characteristic phenotype of *Salmonella*, which typically appears as red colonies with or without black centres resulting from H_2_S production, and were therefore regarded as presumptive isolates.

Only one artisanal sample produced a colony with morphology suggestive of *Salmonella* spp. The isolate was subjected to confirmation according to EN ISO 6579-1, including a slide agglutination test using *Salmonella* antiserum (BulBio, Bulgaria). No agglutination reaction was observed, and the isolate was therefore not confirmed as *Salmonella* spp.

No presumptive *Salmonella* colonies were observed in any of the 50 industrial cheese samples (Table 6). Following completion of the confirmation procedure, all samples were classified as negative for *Salmonella* spp., demonstrating compliance with the microbiological criterion requiring the absence of *Salmonella* spp. in 25 g of ready-to-eat food, as specified in Commission Regulation (EC) No. 2073/2005.

**Table 6:** Detection of *Salmonella* spp. in artisanal and industrial white brined cow’s milk cheese.

Parameter	Artisanal (n = 50)	Industrial (n = 50)	Total
Samples with atypical colonies on XLD agar	7	0	7
Samples with typical presumptive colonies	1	0	1
Confirmed *Salmonella* spp. isolates	0	0	0

### 3.4. Detection of Listeria monocytogenes

During the analysis of 100 samples of white brined cow’s milk cheese, *Listeria monocytogenes* was not detected in any sample.

Following selective enrichment and inoculation onto ALOA, no colonies exhibiting the characteristic morphology of *L. monocytogenes* were observed. Consequently, no confirmatory testing was required (Table 7).

All artisanal and industrial cheese samples were therefore classified as negative for *L. monocytogenes*, demonstrating compliance with the microbiological criterion requiring the absence of *L. monocytogenes* in 25 g of ready-to-eat food, as specified in Commission Regulation (EC) No. 2073/2005.

**Table 7:** Detection of *Listeria monocytogenes* in artisanal and industrial white brined cow’s milk cheese.

Parameter	Artisanal (n = 50)	Industrial (n = 50)	Total
Samples with presumptive colonies on ALOA	0	0	0
Confirmed *Listeria monocytogenes*	0	0	0

The comprehensive microbiological assessment demonstrated that all analyzed cheese samples complied with the European food safety criteria for *Salmonella* spp. and *Listeria monocytogenes*. The low occurrence of β-glucuronidase-positive *Escherichia coli* (2/100 samples) and the absence of confirmed coagulase-positive staphylococci further indicate a high level of production hygiene and microbiological quality, irrespective of the production system.

### 3.5. Yeast Microbiota

The occurrence of yeasts was investigated in all 100 white brined cow’s milk cheese samples included in the study. Yeast growth was detected in 99 samples (99.0%). All artisanal cheeses (50/50; 100%) yielded yeast isolates, whereas among the industrial cheeses, yeasts were recovered from 49 of the 50 samples (98.0%). No yeast growth was detected in only one industrial cheese sample under the cultivation conditions applied.

A total of nine yeast taxa were identified by MALDI-TOF MS. Several cheese samples contained mixed yeast populations comprising two or more species, whereas others yielded a single yeast isolate. The predominant species in artisanal cheeses was *Torulaspora delbrueckii*, while *Debaryomyces hansenii* predominated among industrial cheeses. Other identified yeasts included *Saccharomyces cerevisiae*, *Candida lambica*, *Candida sphaerica* (*Kluyveromyces lactis*), *Candida zeylanoides*, *Candida valida*, *Geotrichum candidum* and *Rhodotorula* spp. The frequency of isolation of the identified yeast species is presented in Table 8.

Statistically significant differences in the frequency of isolation were observed for *Torulaspora delbrueckii*, *Debaryomyces hansenii* and *Saccharomyces cerevisiae*, whereas the distribution of the remaining yeast species did not differ significantly between artisanal and industrial cheeses (Table 8).

Marked differences in the distribution of the predominant yeast species were observed between the two production systems. *Torulaspora delbrueckii* was the dominant yeast species in artisanal cheeses, whereas *Debaryomyces hansenii* predominated in industrial products. In contrast, the remaining yeast species occurred sporadically and at considerably lower frequencies in both cheese groups.

Overall, the identified yeast species represent the typical fungal microbiota associated with white brined cow’s milk cheese and provide additional information on the microbiological profile of the investigated products.

**Figure 1:**
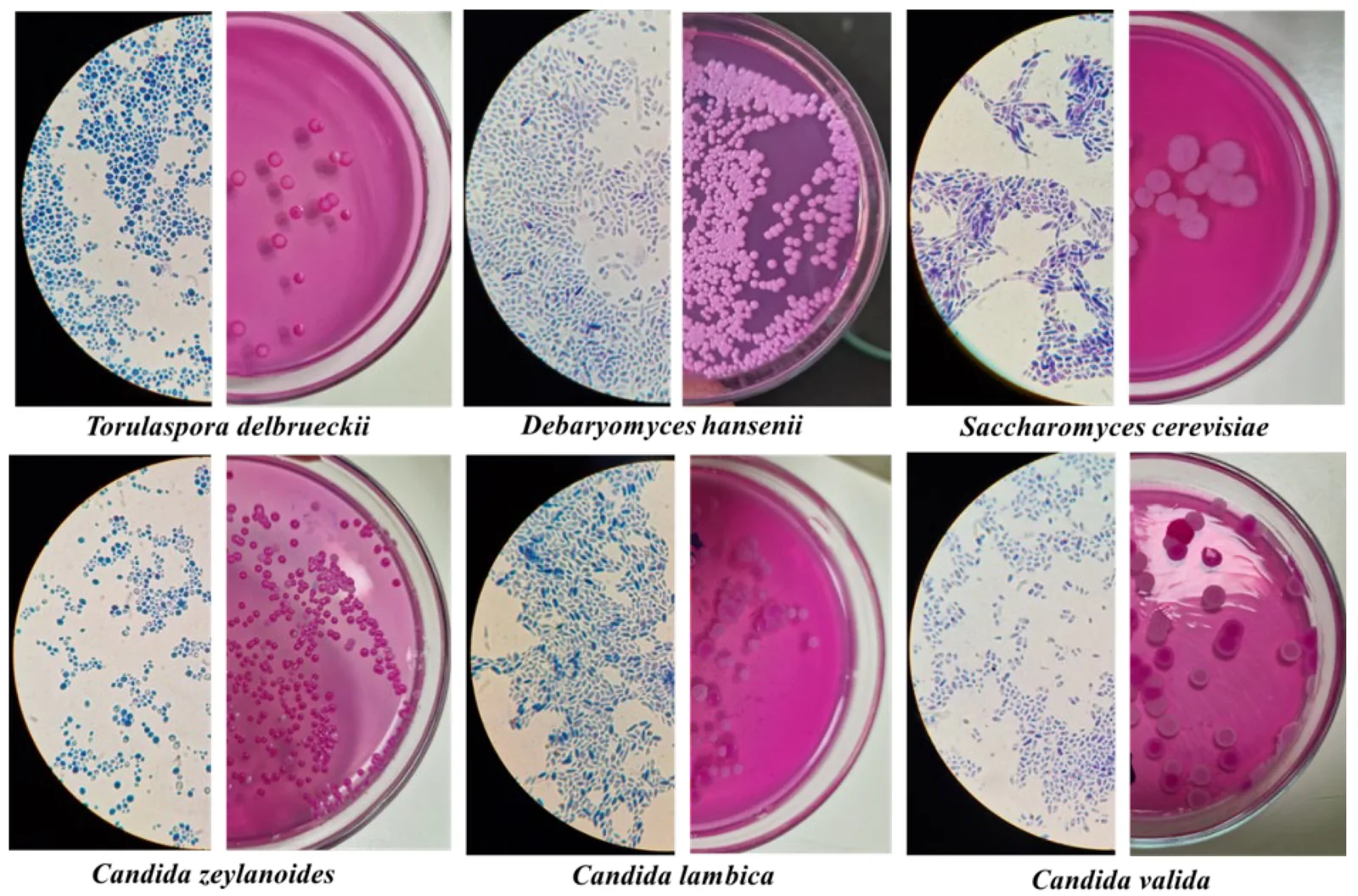
Microscopic images of prepared slides using the Loeffler staining method and colonies on DRBC agar of the isolated yeasts from industrial and artisanal white brined cheeses.

The comparison of yeast species distribution demonstrated distinct differences between artisanal and industrial cheeses. *Torulaspora delbrueckii* was the predominant species in artisanal products, whereas *Debaryomyces hansenii* predominated in industrial cheeses. The remaining yeast species were isolated less frequently and were detected in both production types with variable occurrence. Overall, the results demonstrated a heterogeneous yeast microbiota in the investigated white brined cheeses while confirming the predominance of only a limited number of species (Figure 1).

## 4. Discussion

### 4.1. An Integrated Assessment Provides a More Comprehensive Characterization of Microbiological Safety

The microbiological safety of white brined cheese is traditionally assessed through the detection of major foodborne pathogens [24,25,26]. However, such an approach does not adequately reflect production hygiene, technological characteristics, or the natural microbial ecology of the product. Therefore, the present study applied an integrated approach comprising the analysis of hygiene indicator microorganisms (*Escherichia coli* and coagulase-positive staphylococci), major bacterial pathogens (*Listeria monocytogenes* and *Salmonella* spp.), and the yeast microbiota in 100 samples of Bulgarian white brined cheese, including 50 artisanal and 50 industrial products.

The results demonstrated a favourable microbiological profile irrespective of the production system. *Listeria monocytogenes* and *Salmonella* spp. were not detected in any sample (0/100), no coagulase-positive staphylococci were confirmed, and *Escherichia coli* was detected in only two samples (2.0%), one from each production group, with no statistically significant difference between them (*p* > 0.05). Thus, 98% of the cheeses showed no deviation in any of the investigated bacteriological parameters.

In contrast to the pathogenic microorganisms, yeasts were widely distributed and exhibited substantial species diversity. Within the same sample collection, nine yeast species were identified, with *Debaryomyces hansenii* and *Torulaspora delbrueckii* predominating and displaying distinct distributions between artisanal and industrial cheeses. These findings indicate that a microbiologically safe product is not a sterile environment, but rather a complex microbial ecosystem in which the indigenous microbiota plays an important role in cheese ripening and the development of organoleptic characteristics.

This concept is increasingly supported by recent literature [1,27]. In an analysis of more than 1300 cheeses, Primavilla et al. [28] showed that microbiological non-compliance was associated primarily with hygiene indicators rather than with the major foodborne pathogens. Our findings extend this concept by demonstrating that the inclusion of the natural yeast microbiota complements conventional safety assessment and provides a more comprehensive characterization of the microbiological quality of the product.

Accordingly, the evaluation of white brined cheese should not be based solely on the absence of foodborne pathogens, but rather on an integrated assessment of food safety, production hygiene, and the natural microbiota, which provide complementary information on its overall microbiological quality.

### 4.2. Hygiene Indicators as Reflections of the Production Process

*Escherichia coli* and coagulase-positive staphylococci are among the principal indicators of production hygiene in dairy products, reflecting potential faecal contamination and the risk of staphylococcal contamination, respectively. Their combined assessment provides a more reliable evaluation of production hygiene than the interpretation of either indicator alone.

In the present study, *Escherichia coli* was detected in only 2 of the 100 samples (2.0%), with one positive sample identified among artisanal cheeses (1/50; 2.0%) and one among industrial cheeses (1/50; 2.0%), showing no statistically significant difference between the two groups (*p* > 0.05). In both cases, the organism was recovered only from the first decimal dilution, indicating a low level of contamination. The equal distribution of positive samples suggests that these findings represent sporadic contamination events rather than systematic differences between the production systems. Similar observations have been reported for other traditional cheeses, where the occurrence of *E. coli* is primarily associated with raw milk quality, the effectiveness of hygienic control measures, and the prevention of post-processing contamination [4,29]. Accordingly, the present findings indicate that both artisanal and industrial producers can achieve a high level of production hygiene through the implementation of good manufacturing practices.

Even more informative were the results obtained for staphylococci. Presumptive staphylococcal colonies with characteristic morphology on Baird–Parker agar were observed in 49% of the samples (28 artisanal and 21 industrial cheeses), with no statistically significant difference between the two groups (*p* > 0.05). However, none of the isolates was confirmed as coagulase-positive following the coagulase test, effectively excluding the presence of *Staphylococcus aureus* in the analyzed cheeses.

These findings demonstrate that colony morphology alone is insufficient for reliable risk assessment, as coagulase-negative staphylococci may produce colonies resembling those of *S. aureus* on selective media. Consequently, the confirmatory procedures prescribed by ISO 6888-1 are essential for the accurate evaluation of microbiological safety and prevent overestimation of the risk based solely on presumptive colony morphology.

### 4.3. Significance of the Absence of Major Foodborne Pathogens

*Listeria monocytogenes* and *Salmonella* spp. are among the most important foodborne pathogens associated with ready-to-eat dairy products; therefore, their absence represents a fundamental criterion for microbiological safety [30,31].

In the present study, none of the 100 cheese samples tested positive for *Listeria monocytogenes* or *Salmonella* spp., irrespective of the production system. During the analysis for *Salmonella*, seven artisanal cheese samples exhibited atypical growth on the selective medium, while one sample produced presumptive *Salmonella*-like colonies. However, following serological confirmation, all isolates were identified as negative, highlighting the importance of the complete ISO confirmation procedure and demonstrating that interpretation based solely on colony morphology may lead to false-positive results.

These findings indicate that both artisanal and industrial cheeses complied with the European microbiological safety criteria. Similar observations have been reported for other traditional brined cheeses [13,32], whereas EFSA data indicate that, despite the relatively low prevalence of contamination, ready-to-eat dairy products remain among the food categories of greatest public health concern in sporadic cases of *Listeria monocytogenes* contamination [33]. Therefore, the negative findings reported in the present study should be regarded as evidence of a favourable microbiological status, but not as a justification for reducing routine surveillance of these pathogens.

### 4.4. Yeast Microbiota as a Component of the Microbiological Quality and Technological Characteristics of White Brined Cheese

Unlike bacterial pathogens, yeasts constitute a natural component of the microbial ecosystem of fermented dairy products. Their presence alone should not be interpreted as an indicator of either microbiological safety or spoilage. Depending on the species, strain, abundance, and environmental conditions, yeasts may contribute positively to cheese ripening and sensory development, whereas others may be associated with undesirable metabolic activity and spoilage [34,35].

In the present study, yeasts were detected in 99 of the 100 cheese samples (99.0%), with all artisanal cheeses (50/50) and almost all industrial products (49/50) harbouring viable yeast microbiota. MALDI–TOF MS identified nine yeast taxa, and the species composition differed significantly between the two production systems. Torulaspora delbrueckii predominated in artisanal cheeses (84% vs. 50%; *p* < 0.001), whereas *Debaryomyces hansenii* was characteristic of industrial products (52% vs. 2%; *p* < 0.001). *Saccharomyces cerevisiae* was also isolated more frequently from artisanal cheeses (30% vs. 12%; *p* = 0.048).

These findings indicate that the principal difference between artisanal and industrial cheeses lies not in the prevalence of yeasts but in their species composition. Similar observations have been reported for other traditional cheeses [36,37], where technological factors—including milk pasteurization, starter cultures, ripening conditions, and brine characteristics—have a greater influence on microbial community structure than on the overall abundance of yeasts.

The predominance of *Debaryomyces hansenii* in industrial cheeses is likely related to its pronounced halotolerance and its ability to adapt to standardized technological conditions. In contrast, the higher prevalence of *Torulaspora delbrueckii* and *Saccharomyces cerevisiae* in artisanal cheeses probably reflects a richer indigenous microbiota and the greater influence of the production environment.

Our previous findings further demonstrated that salt concentration is the principal ecological factor shaping yeast species composition, with *D. hansenii* and *T. delbrueckii* exhibiting the highest tolerance to elevated NaCl concentrations. The significant association between salt content and yeast counts observed only in industrial cheeses (*p* = 0.029) suggests that technological selection is more pronounced under standardized production conditions [22]. The same set of 100 cheese samples was also subjected to physicochemical characterization, including salt content, titratable acidity, and degree of maturity, the results of which have been reported separately 38.

From a practical perspective, these findings demonstrate that yeasts should not be regarded solely as agents of microbiological spoilage. Many of the identified species contribute to flavour development and cheese ripening, making the natural yeast microbiota an integral component of the quality of traditional white brined cheese. Consequently, the analysis of yeast communities complements the information provided by foodborne pathogens and hygiene indicators, allowing a more comprehensive assessment of the microbiological quality of the product.

The indigenous yeast isolates identified in this study may represent a valuable microbial resource; however, their potential application as starter or adjunct cultures cannot be inferred from species-level identification alone. Further strain-level characterization, including assessment of relevant enzymatic activities, salt and acid tolerance, aroma-producing capacity, and safety properties, would be required to evaluate their technological potential.

### 4.5. Towards an Integrated Concept for Microbiological Quality Assessment of Traditional White Brined Cheese

The present study demonstrates that traditional artisanal cheese production is not inherently associated with an increased microbiological risk when manufacturing is conducted in accordance with good manufacturing and hygienic practices. Despite harbouring a richer and more diverse yeast microbiota, artisanal cheeses did not differ significantly from industrial products with respect to *Escherichia coli*, coagulase-positive staphylococci, *Listeria monocytogenes*, or *Salmonella* spp. These findings indicate that both production systems are capable of achieving a comparably high level of microbiological safety.

The results further highlight that the natural microbiota should be considered as a component of the overall microbial ecology of the product rather than as a direct indicator of either microbiological safety or spoilage. The greater yeast diversity observed in artisanal cheeses most likely reflects the characteristics of traditional manufacturing practices and the local microbial ecology, whereas the absence of major foodborne pathogens and the low occurrence of hygiene indicator microorganisms demonstrate that this diversity is not associated with an increased microbiological risk.

The practical significance of the present study lies in the application of an integrated approach combining the assessment of foodborne pathogens, hygiene indicators, and the natural yeast microbiota. Our findings demonstrate that this strategy provides a substantially more comprehensive evaluation of the microbiological quality of traditional white brined cheese than the assessment of individual microbiological parameters alone.

Although the present investigation represents one of the largest microbiological surveys of Bulgarian white brined cheese conducted to date (100 samples), season of production was not systematically recorded as a controlled variable for individual samples; therefore, the present dataset does not allow a reliable assessment of potential seasonal variation. Information on the heat treatment of the milk used for individual artisanal cheese samples was not systematically documented, which limited the assessment of its potential influence on microbial composition. Furthermore, while the standardized ISO methods employed provide a reliable assessment of microbiological safety, they do not characterize the overall bacterial community structure or the genetic diversity of individual isolates. Culture-independent approaches, including next-generation sequencing, could complement these methods in future studies by providing a more comprehensive characterization of the cheese microbiome, particularly its lactic acid bacterial communities. However, DNA-based NGS alone cannot establish microbial viability or specifically identify microorganisms in the VBNC state, which would require appropriate viability-oriented approaches. Nevertheless, these limitations do not affect the principal conclusion of the present study. Microbiological safety and a rich indigenous microbial diversity should not be regarded as mutually exclusive characteristics of traditional white brined cheese. Rather, when production is carried out under appropriate hygienic conditions, both can coexist, supporting the concept that the microbiological quality of traditional fermented dairy products should be assessed through an integrated evaluation of food safety, production hygiene, and microbial ecology, rather than solely through the absence of pathogenic microorganisms.

## 5. Conclusions

The present study provides a comprehensive assessment of the microbiological quality and safety of Bulgarian white brined cheese based on 100 artisanal and industrial samples. *Listeria monocytogenes*, *Salmonella* spp., and coagulase-positive staphylococci were not detected, while *Escherichia coli* occurred only sporadically and at low levels, with no significant differences between the two production systems. These findings indicate that artisanal production is not inherently associated with increased microbiological risk when appropriate manufacturing and hygienic practices are maintained.

At the same time, the cheeses harboured a diverse yeast microbiota, with distinct species distributions between artisanal and industrial products, demonstrating that microbial diversity can coexist with a favourable microbiological safety profile. Overall, combining foodborne pathogen detection, hygiene indicators, and characterization of the indigenous microbiota provides a more comprehensive assessment of the microbiological quality of traditional fermented dairy products than pathogen-oriented evaluation alone.

## Figures and Tables

**Table 1:** Prevalence of *Escherichia coli* in artisanal and industrial white brined cow’s milk cheese.

Production Type	Number of Samples	Positive Samples	Prevalence (%)
Artisanal	50	1	2.0
Industrial	50	1	2.0
Total	100	2	2.0

**Table 2:** Prevalence of presumptive *Staphylococcus* spp. in artisanal and industrial white brined cow’s milk cheeses.

Parameter	Artisanal (*n* = 50)	Industrial (*n* = 50)	Total
Positive samples, n (%)	28 (56.0)	21 (42.0)	49 (49.0)
Negative samples, n (%)	22 (44.0)	29 (58.0)	51 (51.0)
Statistical comparison			Fisher’s exact test, *p* = 0.230

**Table 3:** Quantitative distribution of presumptive *Staphylococcus* spp. in positive cheese samples.

Parameter	Artisanal (*n* = 28)	Industrial (*n* = 21)
Mean ± SD (log_10_ CFU/g)	2.90 ± 0.55	3.18 ± 0.42
Median (log_10_ CFU/g)	2.90	3.30
IQR	2.49–3.40	3.00–3.42
Minimum	2.00	2.00
Maximum	3.74	3.91
Statistical comparison	Mann–Whitney U = 214.0; *p* = 0.108	

IQR—interquartile ranges.

**Table 4:** Distribution of presumptive staphylococcal counts according to bacterial concentration.

Bacterial Concentration (log_10_ CFU/g)	Artisanal (n = 28)	Industrial (n = 21)
2.00–2.49	7 (25.0%)	2 (9.5%)
2.50–2.99	7 (25.0%)	3 (14.3%)
3.00–3.49	10 (35.7%)	13 (61.9%)
≥3.50	4 (14.3%)	3 (14.3%)

**Table 5:** Confirmation of presumptive *Staphylococcus* isolates by plasma coagulase test.

Parameter	Number
Samples positive on Baird–Parker agar	49
Representative isolates tested	49
Plasma coagulase-positive isolates	0
Plasma coagulase-negative isolates	49

**Table 8:** Distribution of yeast species in artisanal and industrial Bulgarian white brined cow’s milk cheese.

Yeast Species	Artisanal Cheeses (n = 50)	Industrial Cheeses (n = 50)	*p*-Value
*Torulaspora delbrueckii*	42 (84.0%)	25 (50.0%)	<0.001
*Debaryomyces hansenii*	1 (2.0%)	26 (52.0%)	<0.001
*Saccharomyces cerevisiae*	15 (30.0%)	6 (12.0%)	0.048
*Candida lambica*	7 (14.0%)	3 (6.0%)	0.318
*Candida sphaerica* (*Kluyveromyces lactis*)	6 (12.0%)	9 (18.0%)	0.577
*Candida zeylanoides*	2 (4.0%)	2 (4.0%)	1.000
*Geotrichum candidum*	2 (4.0%)	1 (2.0%)	1.000
*Candida valida*	1 (2.0%)	1 (2.0%)	1.000
*Rhodotorula* spp.	0 (0.0%)	1 (2.0%)	1.000

*p*-values were calculated using Fisher’s exact test.

## Data Availability

The original contributions presented in this study are included in the article. Further inquiries can be directed to the corresponding author.
